# Supplementary material for: Spinal Pain, Chronic Health Conditions and Health Behaviors: Data from the 2016–2018 National Health Interview Survey
Source: Int J Environ Res Public Health. 2023 Apr 3;20(7):5369. doi: 10.3390/ijerph20075369 (PMC10094294; doi:10.3390/ijerph20075369)
Supplement: Supplementary file 1 [file ijerph-20-05369-s001.zip › ijerph-2223740-supplementary.pdf]

Table S1: Data flow chart

Year	Person Module	Sample Adult Module	Functioning and Disability Module	Merged
2016	97,169	33,028	16,478	16,478
2017	78,132	26,742	13,325	13,325
2018	72,831	25,417	25,417	25,417
Total size of the appended study population				55,220
Total size of the final study population, excluding cases with missing values				26,926

Table S2: Distribution of health behaviors, and functional disability, cognitive impairment and mental health conditions by spinal pain status in the US adult population, between 2016-2018

Health behavior	General population		No spinal pain		Neck pain only		Low back pain only		Spinal pain	
	%	95% CI	%	95% CI	%	95% CI	%	95% CI	%	95% CI
Current smokers										
No	84.9	(84.4,85.3)	86.1	(85.7,86.5)	84.7	(83.4,86.0)	80.7	(79.9,81.5)	75.5	(74.4,76.5)
Yes	15.1	(14.0,15.6)	13.9	(13.5,14.3)	15.3	(14.0,16.6)	19.3	(18.5,20.1)	24.5	(23.5,25.6)
Leisure-time physical activity										
Physically inactive	27.6	(26.7,28.6)	26.5	(25.5,27.5)	24.4	(22.6,26.1)	32.2	(30.9,33.4)	35.9	(34.5,37.4)
Insufficiently active	20.2	(26.7,28.6)	19.9	(19.4,20.4)	23.0	(21.4,24.6)	22.0	(21.2,22.8)	22.7	(21.7,23.6)
Sufficiently active	52.2	(26.7,28.6)	53.6	(52.7,54.5)	52.6	(50.7,54.5)	45.8	(44.7,47.0)	41.4	(40.1,42.7)
Sleep problems										
No	62.7	(62.1,63.3)	66.47	(65.9,67.1)	52.8	(50.9,54.6)	52.3	(51.2,53.3)	34.4	(33.2,35.6)
Yes	37.3	(36.7,37.9)	33.53	(32.9,34.1)	47.3	(45.4,49.1)	47.7	(46.7,48.8)	65.6	(64.4,66.8)
Functional disability										
No	77.8	(77.3,78.4)	81.6	(81.1,82.1)	73.5	(71.9,75.0)	66.2	(65.2,67.2)	49.7	(48.4,51.0)
Yes	22.2	(21.6,22.7)	18.4	(17.9,18.9)	26.5	(25.0,28.1)	33.8	(32.8,34.8)	50.3	(49.0,51.6)
Cognitive impairment										
No	97.6	(97.4,97.7)	98.2	(98.1,98.3)	97.4	(96.7,98.1)	96.9	(96.6,97.3)	92.9	(92.2,93.6)
Yes	2.5	(2.3,2.6)	1.8	(1.7,2.0)	2.6	(1.9,3.4)	3.1	(27,3.4)	7.1	(6.4,7.8)
Depression/anxiety										
No	87.0	(86.5,87.4)	89.0	(81.1,82.1)	82.9	(81.2,84.6)	83.3	(82.5,84.2)	71.6	(70.3,72.9)
Yes	13.1	(12.6,13.5)	11.0	(17.9,18.9)	17.1	(154,18.8)	16.7	(15.8,17.5)	28.4	(27.1,29.8)

Table S3. Three models for the associations between cardiovascular conditions and spinal pain variables, in the US adult population, 2016-2018

Chronic health conditions and confounders	No spinal pain	Neck pain only			Low back pain only			Spinal pain		
		OR	95% CI	P	OR	95% CI	P	OR	95% CI	P
**Model 1***										
Cardiovascular conditions	Reference	1.49	(1.35,1.64)	<0.001	1.58	(1.34,1.86)	<0.001	2.41	(2.16,2.68)	<0.001
**Model 2****										
Cardiovascular conditions	Reference	1.45	(1.23,1.72)	<0.001	1.33	(1.21,1.47)	<0.001	1.98	(1.77,2.23)	<0.001
Health behaviors										
Current smokers		1.17	(1.02,1.35)	0.03	1.39	(1.29,1.50)	<0.001	1.54	(1.4,1.70)	<0.001
Leisure-time physical activity										
Physically inactive		0.95	(0.81,1.11)	0.501	1.15	(1.06,1.25)	<0.001	1.13	(1.01,1.26)	0.028
Insufficiently active		1.17	(1.02,1.34)	0.024	1.11	(1.03,1.2)	0.01	1.28	(1.17,1.40)	<0.001
Sufficiently active		Reference			Reference			Reference		
Sleep problems		1.92	(1.72,2.15)	<0.001	1.87	(1.76,1.99)	<0.001	3.44	(3.17,3.74)	<0.001
**Model 3*****										
Cardiovascular conditions	Reference	1.27	(1.02,1.58)	0.03	1.15	(1.00,1.31)	0.05	1.58	(1.35,1.85)	<0.001
Health behaviors										
Current smokers		1.08	(0.90,1.30)	0.386	1.37	(1.25,1.51)	<0.001	1.46	(1.28,1.65)	<0.001
Leisure-time physical activity										
Physically inactive		0.92	(0.76,1.12)	0.417	1.05	(0.95,1.17)	0.32	0.94	(0.82,1.08)	0.367
Insufficiently active		1.10	(0.92,1.31)	0.285	1.11	(1.01,1.21)	0.04	1.19	(1.06,1.33)	0.002
Sufficiently active		Reference			Reference			Reference		
Sleep problems		1.81	(1.58,2.07)	<0.001	1.70	(1.57,1.84)	<0.001	2.80	(2.52,3.12)	<0.001
Functional disability		2.01	(1.62,2.48)	<0.001	2.61	(2.31,2.94)	<0.001	4.24	(3.67,4.89)	<0.001
Cognitive impairment		1.45	(0.77,2.73)	0.246	1.06	(0.71,1.59)	0.76	1.90	(1.35,2.67)	<0.001
Anxiety		1.51	(1.22,1.87)	<0.001	1.39	(1.23,1.58)	<0.001	1.76	(1.53,2.03)	<0.001
Depression		1.36	(1.02,1.82)	0.039	1.24	(1.02,1.50)	0.03	1.70	(1.41,2.05)	<0.001

* Model 1 estimates adjusted for demographic factors (age, sex, and race/ethnicity) and socioeconomic factors (education and earnings)
** Model 2 estimates adjusted for demographic and socioeconomic factors and health behaviors (tobacco use, leisure time physical activity, and sleep problems)
*** Model 3 estimates adjusted for demographic and socioeconomic factors, health behaviors, and functional disability, cognitive impairment, depression, and anxiety

Table S4. Three models for the associations between hypertension and spinal pain variables, in the US adult population, 2016-2018

Chronic health conditions and confounders	No spinal pain	Neck pain only			Low back pain only			Spinal pain		
		OR	95% CI	P	OR	95% CI	P	OR	95% CI	P
**Model 1***										
Hypertension	Reference	1.49	(1.39,1.59)	<0.001	1.32	(1.17,1.49)	<0.001	1.73	(1.59,1.88)	<0.001
**Model 2****										
Hypertension	Reference	1.26	(1.12,1.43)	<0.001	1.40	(1.3,1.5)	<0.001	1.53	(1.4,1.66)	<0.001
Health behaviors										
Current smokers		1.18	(1.03,1.36)	0.022	1.40	(1.3,1.51)	<0.001	1.56	(1.41,1.72)	<0.001
Leisure-time physical activity										
Physically inactive		0.94	(0.8,1.1)	0.43	1.13	(1.04,1.23)	0.004	1.12	(1.01,1.25)	0.037
Insufficiently active		1.16	(1.01,1.33)	0.033	1.10	(1.02,1.18)	0.016	1.26	(1.15,1.38)	<0.001
Sufficiently active		Reference			Reference			Reference		
Sleep problems		1.93	(1.73,2.16)	<0.001	1.86	(1.75,1.98)	<0.001	3.47	(3.2,3.77)	<0.001
**Model 3*****										
Hypertension	Reference	1.23	(1.05,1.43)	0.01	1.31	(1.2,1.43)	<0.001	1.40	(1.25,1.56)	<0.001
Health behaviors										
Current smokers		1.09	(0.91,1.3)	0.344	1.38	(1.25,1.51)	<0.001	1.46	(1.29,1.65)	<0.001
Leisure-time physical activity										
Physically inactive		0.91	(0.75,1.11)	0.35	1.04	(0.94,1.15)	0.455	0.93	(0.81,1.07)	0.329
Insufficiently active		1.09	(0.91,1.3)	0.346	1.09	(0.99,1.2)	0.071	1.17	(1.05,1.31)	0.005
Sufficiently active		Reference			Reference			Reference		
Sleep problems		1.81	(1.58,2.07)	<0.001	1.69	(1.57,1.83)	<0.001	2.81	(2.53,3.13)	<0.001
Functional disability		2.00	(1.62,2.48)	<0.001	2.54	(2.26,2.87)	<0.001	4.23	(3.67,4.88)	<0.001
Cognitive impairment		1.45	(0.77,2.74)	0.249	1.06	(0.71,1.58)	0.784	1.94	(1.39,2.71)	<0.001
Anxiety		1.51	(1.22,1.86)	<0.001	1.39	(1.23,1.57)	<0.001	1.78	(1.54,2.05)	<0.001
Depression		1.38	(1.03,1.84)	0.031	1.23	(1.02,1.49)	0.03	1.71	(1.42,2.06)	<0.001

* Model 1 estimates adjusted for demographic factors (age, sex, and race/ethnicity) and socioeconomic factors (education and earnings)

** Model 2 estimates adjusted for demographic and socioeconomic factors and health behaviors (tobacco use, leisure time physical activity, and sleep problems)

*** Model 3 estimates adjusted for demographic and socioeconomic factors, health behaviors, and functional disability, cognitive impairment, depression, and anxiety

Table S5. Three models for the associations between diabetes and spinal pain variables, in the US adult population, 2016-2018

Chronic health conditions and confounders	No spinal pain	Neck pain only			Low back pain only			Spinal pain		
		OR	95% CI	P	OR	95% CI	P	OR	95% CI	P
**Model 1***										
Diabetes	Reference	1.41	(1.26,1.57)	<0.001	1.28	(1.05,1.56)	0.013	1.60	(1.40,1.82)	<0.001
**Model 2****										
Diabetes	Reference	1.25	(1.03,1.52)	0.027	1.33	(1.19,1.49)	<0.001	1.41	(1.23,1.62)	<0.001
Health behaviors										
Current smokers		1.19	(1.03,1.37)	0.021	1.40	(1.3,1.51)	<0.001	1.56	(1.41,1.72)	<0.001
Leisure-time physical activity										
Physically inactive		0.93	(0.79,1.09)	0.371	1.13	(1.04,1.23)	0.005	1.13	(1.02,1.27)	0.023
Insufficiently active		1.16	(1.01,1.33)	0.04	1.11	(1.03,1.20)	0.005	1.28	(1.17,1.4)	<0.001
Sufficiently active		Reference			Reference			Reference		
Sleep problems		1.96	(1.75,2.19)	<0.001	1.87	(1.76,1.99)	<0.001	3.54	(3.25,3.84)	<0.001
**Model 3*****										
Diabetes	Reference	1.12	(0.88,1.44)	0.353	1.20	(1.05,1.38)	0.009	1.25	(1.05,1.49)	0.01
Health behaviors										
Current smokers		1.10	(0.92,1.32)	0.29	1.37	(1.24,1.51)	<0.001	1.45	(1.28,1.65)	<0.001
Leisure-time physical activity										
Physically inactive		0.90	(0.74,1.09)	0.283	1.03	(0.93,1.15)	0.537	0.95	(0.82,1.09)	0.424
Insufficiently active		1.08	(0.9,1.29)	0.428	1.11	(1.01,1.22)	0.029	1.20	(1.08,1.35)	0.001
Sufficiently active		Reference			Reference			Reference		
Sleep problems		1.83	(1.59,2.1)	<0.001	1.69	(1.56,1.83)	<0.001	2.83	(2.54,3.16)	<0.001

Functional disability	2.15	(1.75,2.66)	<0.001	2.65	(2.35,2.99)	<0.001	4.29	(3.70,4.96)	<0.001
Cognitive impairment	1.17	(0.62,2.23)	0.63	1.03	(0.69,1.56)	0.869	2.00	(1.42,2.81)	<0.001
Anxiety	1.47	(1.18,1.82)	<0.001	1.41	(1.25,1.59)	<0.001	1.77	(1.53,2.05)	<0.001
Depression	1.34	(0.99,1.81)	0.058	1.23	(1.02,1.48)	0.033	1.72	(1.43,2.08)	<0.001

* Model 1 estimates adjusted for demographic factors (age, sex, and race/ethnicity) and socioeconomic factors (education and earnings).
** Model 2 estimates adjusted for demographic and socioeconomic factors and health behaviors (tobacco use, leisure-time physical activity, and sleep problems).
*** Model 3 estimates adjusted for demographic and socioeconomic factors, health behaviors, and functional disability, cognitive impairment, depression, and anxiety.

Table S6. Three models for the associations between obesity and spinal pain variables, in the US adult population, 2016-2018

Chronic health conditions and confounders	No spinal pain	Neck pain only			Low back pain only			Spinal pain		
		OR	95% CI	P	OR	95% CI	P	OR	95% CI	P
Model 1*										
Obesity	Reference	0.92	(0.81,1.03)	0.15	1.44	(1.36,1.54)	<0.001	1.39	(1.28,1.50)	<0.001
Model 2**										
Obesity	Reference	0.89	(0.79,1.01)	0.061	1.40	(1.32,1.50)	<0.001	1.30	(1.20,1.40)	<0.001
Health behaviors										
Current smokers		1.16	(1.00,1.35)	0.043	1.43	(1.33,1.55)	<0.001	1.60	(1.46,1.77)	<0.001
Leisure-time physical activity										
Physically inactive		0.97	(0.83,1.14)	0.717	1.11	(1.02,1.21)	0.01	1.13	(1.01,1.25)	0.031
Insufficiently active		1.20	(1.04,1.37)	0.012	1.08	(1,10.160)	0.06	1.27	(1.16,1.38)	<0.001
Sufficiently active		Reference			Reference			Reference		
Sleep problems		1.95	(1.74,2.18)	<0.001	1.87	(1.76,1.98)	<0.001	3.52	(3.24,3.82)	<0.001
Model 3***										
Obesity	Reference	0.82	(0.71,0.96)	0.011	1.34	(1.25,1.45)	<0.001	1.17	(1.07,1.28)	0.001
Health behaviors										
Current smokers		1.06	(0.89,1.28)	0.504	1.41	(1.28,1.55)	<0.001	1.50	(1.32,1.70)	<0.001
Leisure-time physical activity										
Physically inactive		0.95	(0.78,1.15)	0.57	1.04	(0.93,1.15)	0.502	0.95	(0.82,1.09)	0.435
Insufficiently active		1.13	(0.95,1.35)	0.178	1.07	(0.97,1.18)	0.163	1.19	(1.06,1.33)	0.003
Sufficiently active		Reference			Reference			Reference		
Sleep problems		1.82	(1.58,2.08)	<0.001	1.70	(1.57,1.83)	<0.001	2.84	(2.54,3.16)	<0.001
Functional disability		2.14	(1.73,2.65)	<0.001	2.47	(2.19,2.79)	<0.001	4.31	(3.73,4.98)	<0.001
Cognitive impairment		1.46	(0.77,2.76)	0.246	1.05	(0.7,1.58)	0.804	1.99	(1.43,2.78)	<0.001
Anxiety		1.48	(1.20,1.84)	<0.001	1.40	(1.24,1.59)	<0.001	1.80	(1.56,2.08)	<0.001
Depression		1.42	(1.06,1.91)	0.019	1.22	(1.01,1.48)	0.042	1.68	(1.39,2.03)	<0.001

* Model 1 estimates adjusted for demographic factors (age, sex, and race/ethnicity) and socioeconomic factors (education and earnings)

** Model 2 estimates adjusted for demographic and socioeconomic factors and health behaviors (tobacco use, leisure-time physical activity, and sleep problems)

*** Model 3 estimates adjusted for demographic and socioeconomic factors, health behaviors, and functional disability, cognitive impairment, depression, and anxiety
